# Further structure–activity relationships study of substituted dithiolethiones as glutathione-inducing neuroprotective agents

**DOI:** 10.1186/s13065-016-0210-z

**Published:** 2016-10-19

**Authors:** Dennis A. Brown, Swati Betharia, Jui-Hung Yen, Ping-Chang Kuo, Hitesh Mistry

**Affiliations:** 1 Department of Pharmaceutical Sciences, Manchester University College of Pharmacy, 10627 Diebold Rd, Fort Wayne, IN 46845 USA; 2 Department of Microbiology and Immunology, Indiana University School of Medicine, 2101 E. Coliseum Blvd, Fort Wayne, IN 46805 USA

**Keywords:** Neuroprotection, Parkinson’s disease, Glutathione, Dithiolethiones

## Abstract

**Background:**

Parkinson’s disease is a neurodegenerative disorder associated with oxidative stress and glutathione depletion. The induction of cellular glutathione levels by exogenous molecules is a promising neuroprotective approach to limit the oxidative damage that characterizes Parkinson’s disease pathophysiology. Dithiolethiones, a class of sulfur-containing heterocyclic molecules, are known to increase cellular levels of glutathione; however, limited information is available regarding the influence of dithiolethione structure on activity. Herein, we report the design, synthesis, and pharmacological evaluation of a further series of dithiolethiones in the SH-SY5Y neuroblastoma cell line.

**Results:**

Our structure–activity relationships data show that dithiolethione electronic properties, given as Hammett σ_p_ constants, influence glutathione induction activity and compound toxicity. The most active glutathione inducer identified, **6a**, dose-dependently protected cells from 6-hydroxydopamine toxicity. Furthermore, the protective effects of **6a** were abrogated by the inhibitor of glutathione synthesis, buthionine sulfoximine, confirming the importance of glutathione in the protective activities of **6a**.

**Conclusions:**

The results of this study further delineate the relationship between dithiolethione chemical structure and glutathione induction. The neuroprotective properties of analog **6a** suggest a role for dithiolethiones as potential antiparkinsonian agents.

## Background

The incidences of neurodegenerative disorders are expected to greatly increase as the American population ages. Parkinson’s disease (PD), the second most common neurodegenerative disease, is a movement disorder characterized by the gradual disintegration of the nigrostriatal dopaminergic pathway. The resulting depletions of striatal dopamine (DA) give rise to the cardinal symptoms of the disease, including tremor, rigidity, bradykinesia, and postural instability. Additionally, cognitive issues, depression, and sleep disturbances are frequently observed non-motor symptoms. Although pharmacotherapeutic intervention is capable of providing symptomatic relief in PD, to date no therapy is able to arrest or reverse the progression of the disease.

**Fig. 1:**
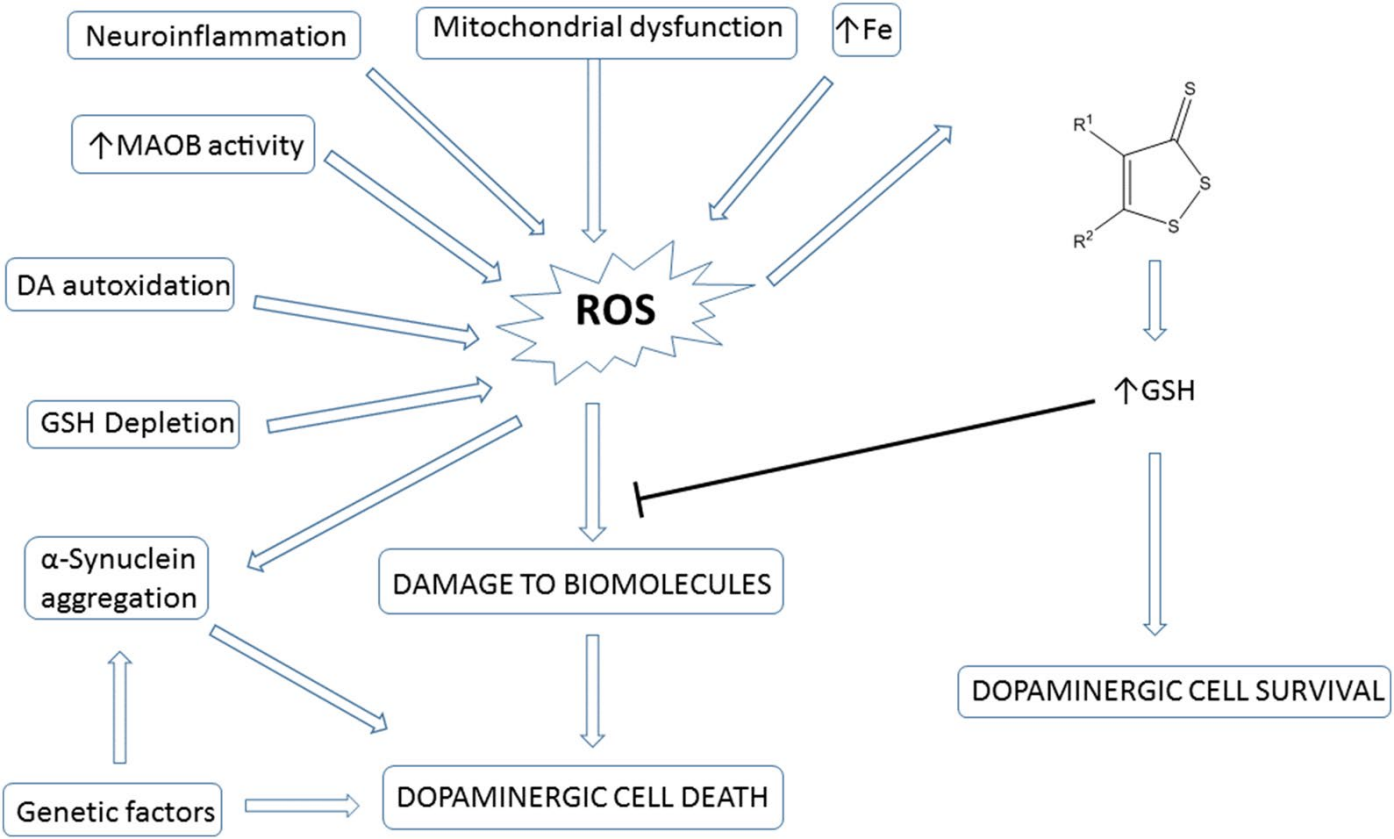
Sources of oxidative stress in PD

The cause of PD is not currently fully understood; however, the etiology of sporadic PD, the most prevalent form of the disease, is probably multifactorial, involving a combination of genetic, environmental, and unknown factors. Increasingly, oxidative stress is emerging as a major player in neurodegenerative disorders such as PD. Analyses of the brains of PD patients have demonstrated extensive cellular damage caused by oxidative stress [1]. Neurons may be particularly prone to oxidative damage due to their high lipid content and oxygen consumption. Dopaminergic neurons experience an additional oxidative burden due to the autoxidation and metabolism of DA. These processes yield damaging electrophilic DA-quinones and reactive oxygen species (ROS). Additionally, many of the molecular hallmarks of PD, such as mitochondrial dysfunction, α-synuclein aggregation, neuroinflammation, increased monoamine oxidase B activity, and elevated levels of iron, are related to increased oxidative activity [2–7]. ROS cause lipid peroxidation, protein and DNA damage, and ultimately the demise of dopaminergic neurons [8–10] (Fig. 1).

As reactive oxygen species occur naturally in all cells, various antioxidants and enzymes have been evolved to mitigate their harmful effects. Glutathione (GSH), a cysteine-containing tripeptide, is the most abundant non-protein antioxidant in the body, and plays a crucial role in the detoxification of ROS and dopamine metabolites [11]. GSH can detoxify ROS non-enzymatically, forming oxidized glutathione (GSSG). GSH also serves as a cosubstrate for several phase II enzymes. Glutathione *S*-transferase (GST) mediates the addition of GSH to electrophiles, such as dopamine *o*-quinone, and glutathione peroxidase (GPx) catalyzes the reduction of peroxides, including H_2_O_2_ [12, 13]. However, in PD, the oxidative load experienced by dopaminergic neurons overwhelms these endogenous cellular detoxification mechanisms. Indeed, postmortem analyses of the brains of PD patients have shown depleted levels of nigrostriatal GSH [14]. As such, increasing neuronal levels of GSH may provide therapeutic benefit against the damaging effects of oxidative stress in PD.

The rate-limiting step in the biosynthesis of GSH is mediated by glutamate cysteine ligase (GCL). Associated with the gene of this enzyme is the antioxidant response element (ARE), found in many genes that play a role in protecting cells from oxidative damage, including GCLC (the catalytic subunit of GCL), GST, GPx, NAD(P)H:quinone oxidoreductase (NQO1), superoxide dismutase, hemeoxygenase, catalase, and many others [15]. Stabilization and nuclear translocation of the transcription factor Nrf2 (nuclear factor-erythroid-2 related factor-2) enhances the transcription of ARE-associated genes [16]. Nrf2 is a short-lived protein, undergoing rapid ubiquitination and proteasomal degradation under basal conditions, mediated by its repressor Keap1 (Kelch-like ECH-associated protein-1) [17–19]. Keap1 is a cysteine-rich protein that serves as a sensor of oxidative and electrophilic stress. The stabilization of Nrf2 is believed to involve modulation of some of the numerous cysteine residues of Keap1 by ROS and electrophiles, leading to enhanced Nrf2 stability and nuclear accumulation [20–22].

**Fig. 2:**
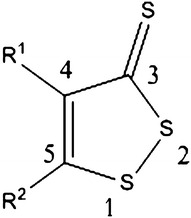
Generalized structure of dithiolethiones

Dithiolethiones (DTTs) are a class of sulfur-containing heterocycles (Fig. 2). DTTs have been shown to induce the expression of a variety of ARE-associated detoxification enzymes and molecules, including GCLC and GSH, in numerous cell and tissue types; however, limited information is available regarding the activities of these interesting molecules in the CNS [23–25]. Our group is interested in exploring GSH induction as a potential neuroprotective strategy. In a previous report by our group, we described a preliminary SAR study of substituted DTTs as inducers of GSH in the SH-SY5Y neuroblastoma cell line (a dopaminergic cell line commonly employed in in vitro models of PD), with key findings that placement of electron withdrawing groups (EWGs) at the 4-position and electron donating groups (EDGs) at the 5-position induced the most glutathione [26–28]. Additionally, three of these GSH inducers demonstrated neuroprotection in the in vitro 6-hydroxydopamine (6-OHDA) model of neurotoxicity. Based on these initial findings, we sought to better understand the influence of DTT substituents on GSH induction. In this report, we describe the synthesis and GSH induction activities of additional substituted DTTs. The relationship between DTT structure and pharmacological activity is discussed.

## Chemistry

**Scheme 1:**
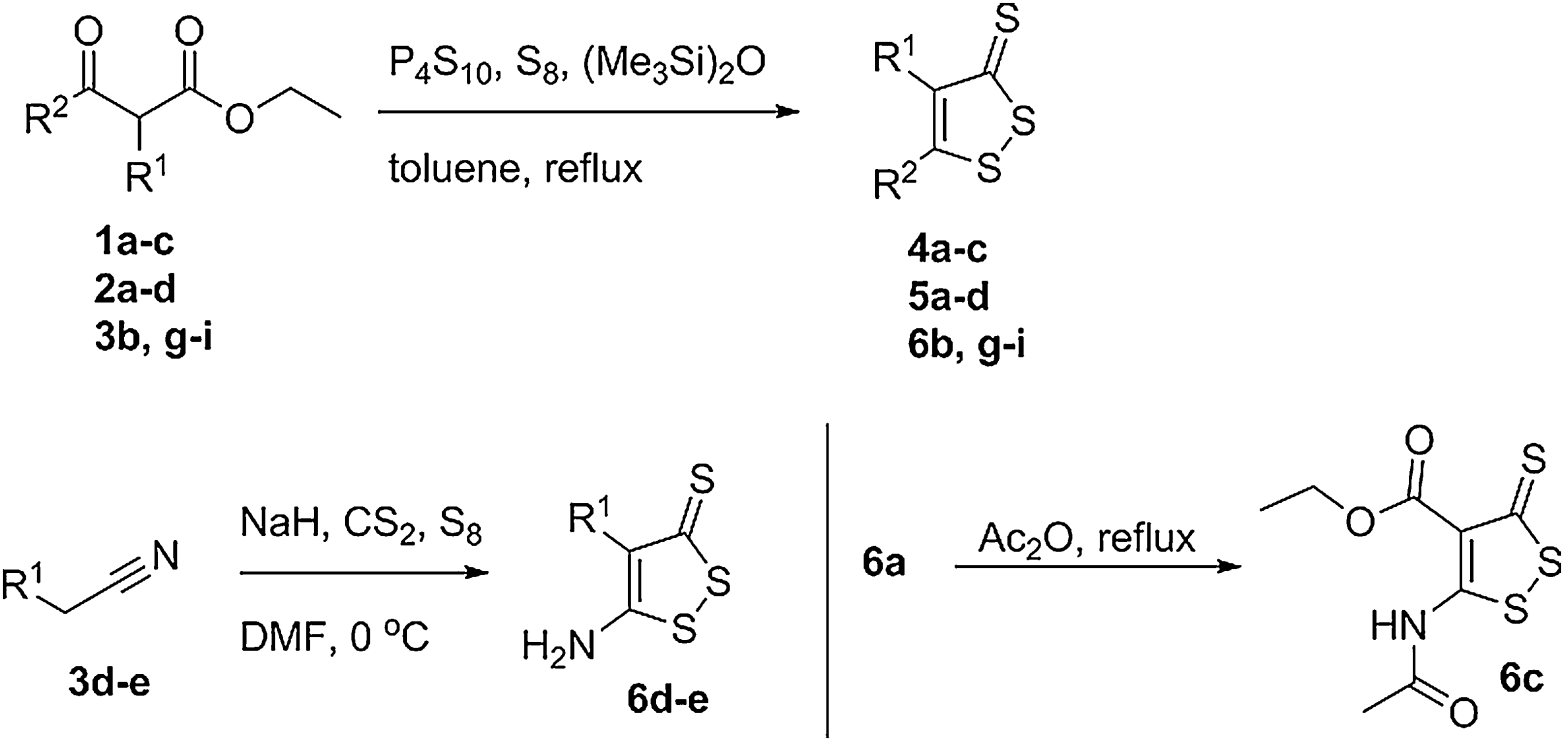
Synthesis of dithiolethiones

A series of 4-, 5-, and 4, 5-disubstituted DTTs was synthesized (Table 1) to determine the generality of the initial SAR findings previously communicated by us [26]. These molecules were designed to ensure that a diversity of electronic features were represented in the compounds evaluated, including various aryl, alkyl, and amino groups, with both electron donating and electron withdrawing properties. The syntheses of DTTs are shown in Scheme 1. Compounds **4a**–**c**, **5a**–**d**, and **6b, g**–**i** were synthesized from requisite β-keto esters by treatment with P_4_S_10_, S_8_, and (Me_3_Si)_2_O in refluxing toluene for 1–3 h in good to excellent yield [29]. Molecules **6d**–**e** were synthesized from their corresponding nitriles via reaction with NaH, S_8_, and CS_2_ in DMF at 0 °C for 30 min, in excellent yield [30]. Compound **6c** was synthesized by refluxing **6a** in acetic anhydride for 30 min (Scheme 1). Molecules **6a** and **6f** were purchased commercially.

**Table 1:** Structures and Hammett sigma constants of DTTs

Entry	R^1^ (σ_p_) [31]	R^2^ (σ_p_) [31]	Entry
–	H	H	**D3T**
**1a**	4-NO_2_-C_6_H_4_ (0.26)	H (0)	**4a**
**1b**	Ethyl (−0.15)	H (0)	**4b**
**1c**	CO_2_Et (0.50)	H (0)	**4c**
**2a**	H (0)	Me (−0.17)	**5a**
**2b**	H (0)	4-F-C_6_H_4_ (0.06)	**5b**
**2c**	H (0)	4-pyridinyl (0.44)	**5c**
**2d**	H (0)	2-furanyl (0.02)	**5d**
**3a**	CO_2_Et (0.50)	NH_2_ (−0.66)	**6a**
**3b**	CO_2_Et (0.50)	Me (−0.17)	**6b**
**3c**	CO_2_Et (0.50)	NHC(O)Me (0.00)	**6c**
**3e**	4-Cl-C_6_H_4_ (0.12)	NH_2_ (−0.66)	**6d**
**3d**	SO_2_Ph (0.68)	NH_2_ (−0.66)	**6e**
**3f**	CN (0.66)	NH_2_ (−0.66)	**6f**
**3g**	Cl (0.23)	4-OMe-C_6_H_4_ (−0.08)	**6g**
**3h**	Cl (0.23)	C_6_H_5_ (−0.01)	**6h**
**3i**	Cl (0.23)	Ethyl (−0.15)	**6i**

## Results and discussion

**Fig. 3:**
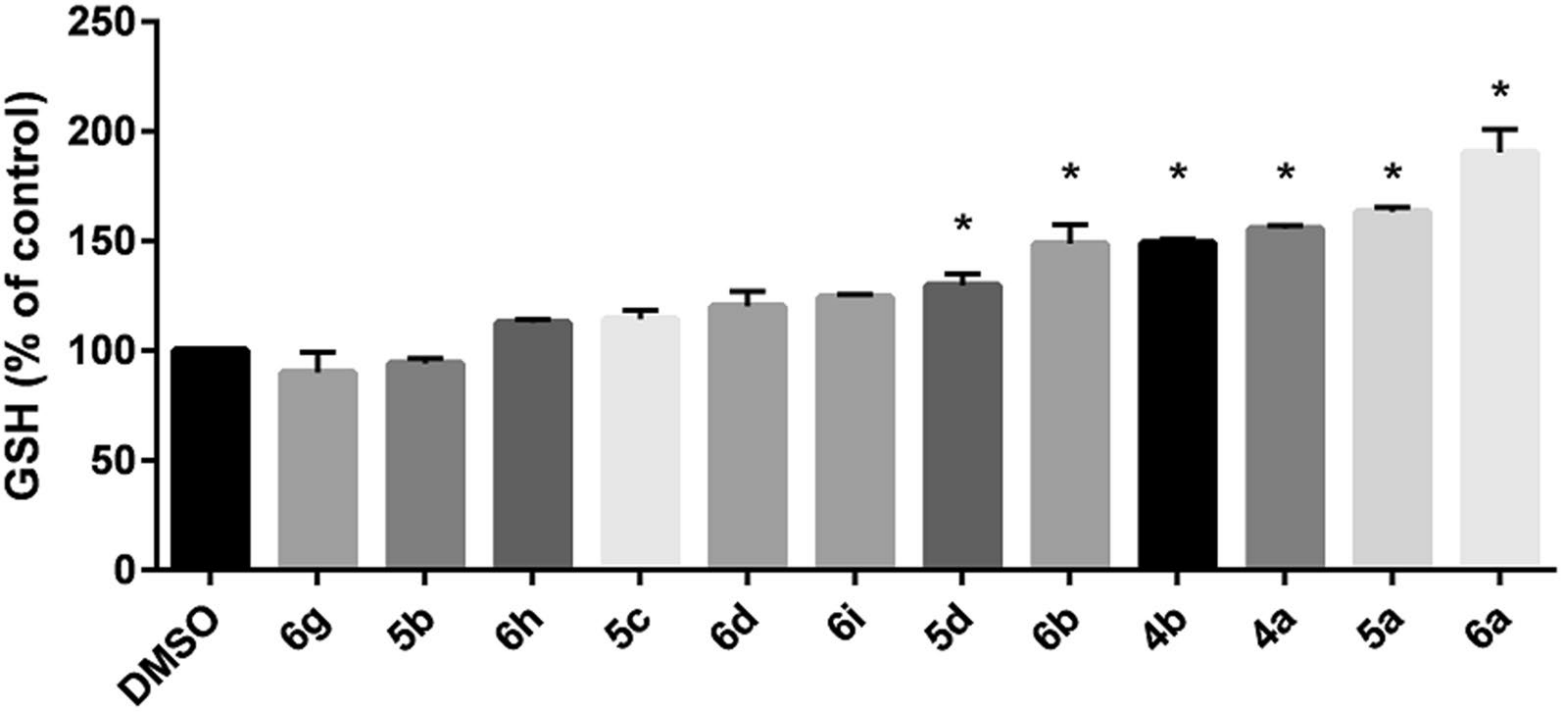
DTT-mediated GSH induction. SH-SY5Y cells were treated with test compounds (100 μM) for 24 h, at which time total cellular GSH was measured. Data shown are mean ± SEM of at least three different experiments. *P < 0.05

DTTs were assayed for GSH induction. SH-SY5Y human neuroblastoma cells were treated with test compounds for 24 h at a concentration of 100 μM. The results are shown in Fig. 3 and are reported as a percentage of control. Among the four 5-substituted DTTs (**5a**–**d**) evaluated, electron-donating 5-methyl substituted DTT **5a** induced GSH to the highest extent (163 %) compared to the other 5-substituted DTTs evaluated. Compounds **5b, 5c,** and **5d**, each containing electron-withdrawing aromatic groups, induced a lesser amount of GSH (94, 114 and 130 %, respectively). These results are consistent with our previous findings that alkyl groups at this position are superior to aromatic groups.

Next evaluated were three 4-substituted molecules, **4a**–**c**, containing *p*-nitrophenyl, ethyl, and ester groups, respectively. Interestingly, electronically-different **4a** and **4b** increased GSH levels by almost the same extent (156 % for **4a**, and 149 % for **4b**). The activity of **4b** is unexpected, as our previous work suggested that EDGs at this position would induce less GSH as their electron-withdrawing counterparts. Surprisingly, when 4-ethyl ester substituted analog **4c** was tested, significant toxicity was observed, and the GSH induction data for this compound was omitted (vide infra).

Next, we explored the effects on GSH induction of substituting both the 4- and 5-positions of the DTT core with a variety of functional groups (compounds **6a**–**i**). The most active molecule in this series was analog **6a** (4-ethyl ester, 5-amino), which increased cellular GSH levels by 190 %. Interestingly, replacement of the primary amine of **6a** with a methyl group, **6b**, significantly reduced activity. Similarly, substitution of the ester of **6a** with an aryl ring (**6d**) or chloro group (**6g**–**i),** diminished activity, regardless of the nature of the 5-position. The SAR data from disubstituted DTTs suggest that GSH induction is highest when the 4- and 5-positions possess strongly electron withdrawing and strongly electron donating groups, respectively. Compounds **6e** (4-phenylsulfonyl, 5-amino) and **6f** (4-nitrile, 5- amino) exhibited toxicity when evaluated and the resulting GSH induction data were omitted (vide infra).

**Fig. 4:**
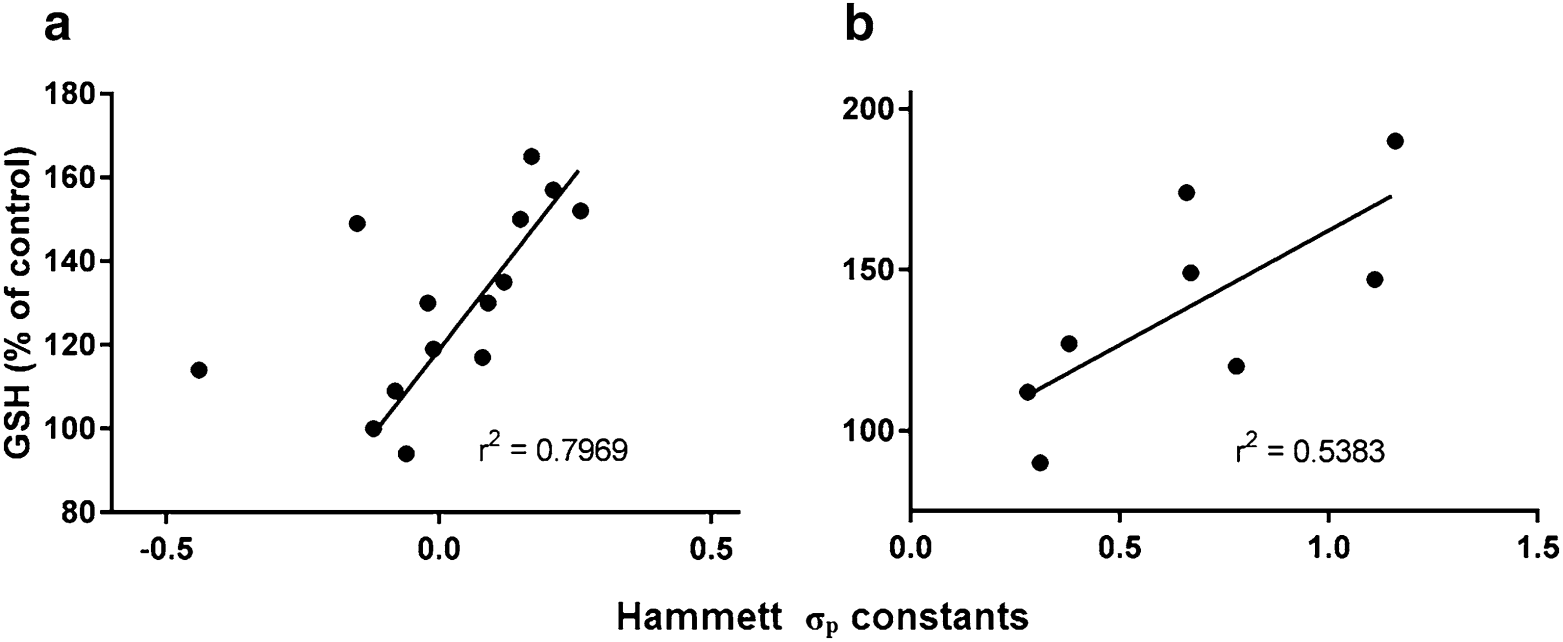
GSH induction values of 4- and 5-substituted DTTs (**a**), and 4, 5-disubstituted DTTs (**b**) vs. Hammett σ_p_ constants

The above SAR data demonstrate that electronic parameters influence GSH induction activity. As such, we sought a method to quantitatively assess the electronic properties of substituted DTTs. We decided to explore Hammett’s σ_p_ constants (Table 1), which reflect the ability of substituted benzoic acids to stabilize a negatively charged carboxylate upon ionization of the corresponding acid. The constants given for these ionizations are an indication of the release (−σ_p_) or withdrawal (+σ_p_) of electrons by a substituent, and provide an indication of the combined contributions of both inductive and resonance effects. We plotted our GSH induction values for 4- and 5-substituted compounds from this and our previous study (structures shown in Table 2) against reported Hammett σ_p_ constants (Fig. 4) [31]. As EDGs at the 5-position were observed to be beneficial to activity, we chose to use +σ_p_ for these types of functional groups, and −σ_p_ for EWGs, which appeared to impair GSH induction. Analogously, as EWGs generally had a positive influence on activity at the 4-position, we used +σ_p_; −σ_p_ were employed for the less active EDGs. As can be seen in Fig. 4a, a linear relationship was observed between DTT electronic properties and GSH induction, with only two molecules, **4b** and **5c**, laying outside of the curve (r^2^ = 0.7969 with **4b** and **5c** omitted). Interested in whether electronics similarly influence activity for the 4, 5-disubstituted molecules, we summed the σ_p_ constants of both substituents (using the same approach to the sign of σ_p_ described above) and plotted these values with the respective GSH activity. Again, a relationship was seen, supporting the influence of electronic properties on GSH induction (r^2^ = 0.5383, Fig. 4b).

**Table 2:** DTT structures from initial SAR study and corresponding Hammett sigma constants [26, 31]

R^1^ (σ_p_)	R^2^ (σ_p_)	Entry
4-OMe-C_6_H_4_ (−0.08)	H (0)	**4d**
C_6_H_5_ (−0.01)	H (0)	**4e**
CH_2_CF_3_ (0.09)	H (0)	**4f**
4-Cl-C_6_H_4_ (0.12)	H (0)	**4g**
H (0)	Ethyl (−0.15)	**5e**
H (0)	Cyclopropyl (−0.21)	**5f**
H (0)	4-Cl-C_6_H_4_ (0.12)	**5g**
H (0)	4-OMe-C_6_H_4_ (−0.08)	**ADT**

**Fig. 5:**
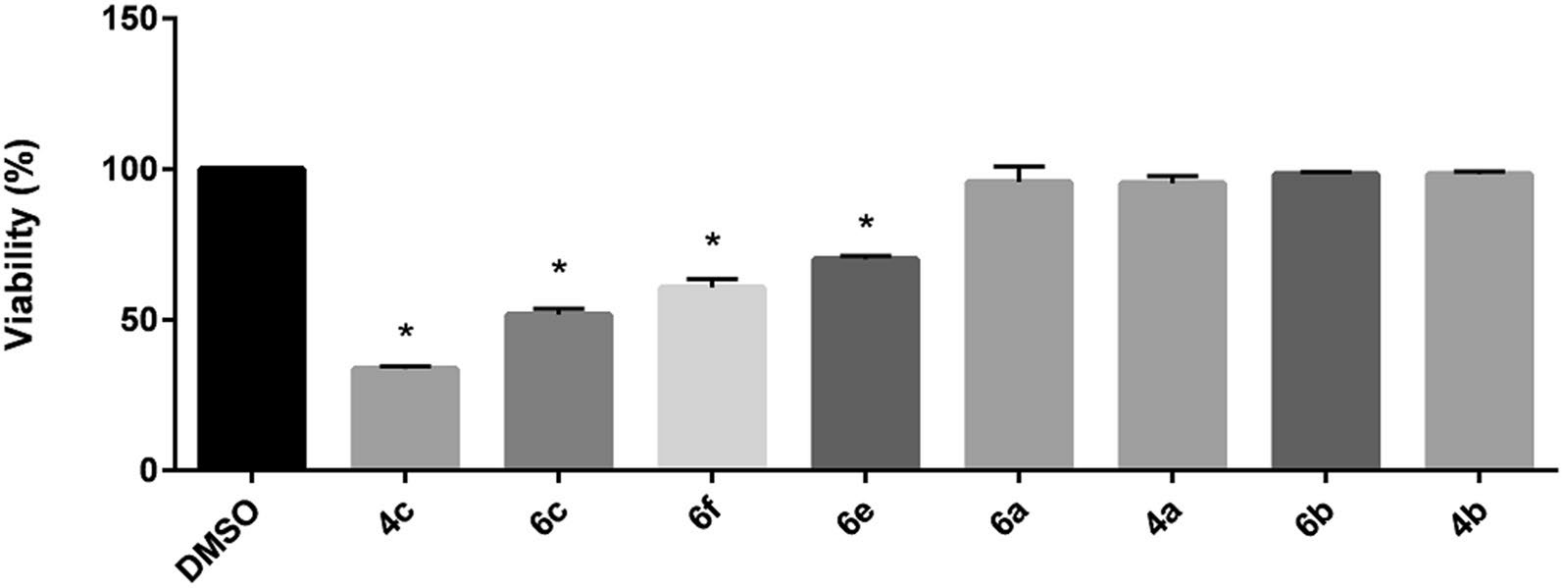
Toxicity of DTTs. SH-SY5Y cells were treated with the indicated molecules (100 μM) for 24 h, at which time viability was assessed. Data shown are mean ± SEM of at least three different experiments. *P < 0.05

As previously mentioned, when DTTs **4c**, **6e**, and **6f** were evaluated for GSH induction in SH-SY5Y cells, significant toxicity was observed, and the GSH induction data for these molecules was omitted from the study. Interestingly, analogs **6a** and **6b**, amino and methyl 5-substituted congeners of **4c**, appeared to not be toxic to SH-SY5Y cells. Based on this observation, we began to suspect that DTT toxicity may be related to the value of σ_p_ at the 4-position. To test this hypothesis, we measured the viability of SH-SY5Y cells treated with our DTTs (100 µM, 24 h, Fig. 5). Molecules with 4-position σ_p_ constants ranging from −0.15 (**4b**) to 0.26 (**4a**) showed minimal toxicity to SH-SY5Y cells. However, when the σ_p_ constant was raised to 0.50 (**4c**), significant cell death was seen. Surprisingly, the addition of an amino or methyl substituent to the 5-position of **4c** (compounds **6a** and **6b**, respectively) appeared to restore viability. To confirm the beneficial effects on toxicity of an electron-donating group at the 5-position, the amino group of **6a** was acylated, yielding **6c**. As the σ_p_ constant of the acetamide group is 0.0, electron donation should not take place, and **6c** would be expected to be toxic. This was indeed observed as shown by the restoration of toxicity of **6c**. The beneficial effects of placing electron-donating substituents at the 5-position appears to be limited, however. When the σ_p_ constant of the 4-position of **6a** (ethyl ester, σ_p_ = 0.50) was increased to 0.66 (nitrile, compound **6f**), or 0.68 (sulfone, compound **6e**), cell viability was once again decreased.

The above observation that GSH induction is dependent on the magnitude of Hammett σ_p_ constants suggests that DTTs substituents influence the reactivity of the dithiolethione ring. Stabilization of Nrf2 by DTTs is believed to result from alteration of the interaction between Nrf2 and its repressor, Keap1. In the presence of oxygen and cellular thiols, the DTTs D3T, oltipraz, and ADT generate superoxide anion, O_2_, a progenitor to H_2_O_2_ [32–34]. Either of these reactive oxygen species could oxidize the numerous sulfhydryl groups of Keap1, resulting in diminished ubiquitination and increased nuclear accumulation of Nrf2. The placement of substituents with larger σ_p_ constants on the dithiolethione ring may render the molecule more reactive to thiols, resulting in greater GSH induction. It is also likely that the toxicity observed by several of the evaluated DTTs may be a consequence of the above described mechanism of action. The DTTs that were observed to be toxic to SH-SY5Y cells (**4c**, **6c**, **6e** and **6f**) would be expected to induce more GSH than other evaluated DTTs, based on extrapolation of our GSH induction vs. σ_p_ plots. Given the current evidence for the proposed mechanism of action of Nrf2 activation by DTTs, it is possible that toxicity results from an increased level of reactive oxygen species produced from DTTs with higher σ_p_ constants for the 4-position. Additional studies are currently planned to more clearly understand the nature of DTT toxicity.

The observed influence of DTT substituent σ_p_ constants on GSH induction and compound toxicity has important implications in the design and selection of future molecules as neuroprotective agents. 4-Monosubstituted congeners must possess substituents with σ_p_ constants that are less than 0.5 to avoid toxicity, thus limiting the extent of GSH induction possible. Their 5-monosubstituted counterparts must have strongly electron-donating groups to effect significant GSH induction; however, aliphatic groups, the most active function group at this position, were only able to increase GSH by a maximum of 165 % (compound **5a**). Substitution of carbon-containing substituents at the 5-position with heteroatoms (O, N) would increase the electron donating effects at this site; however, efforts to synthesize such monosubstituted analogs proved to be problematic. Disubstituted DTT **6a** appears to solve both of these issues: the strongly electron withdrawing ester at the 4-position, combined with the electron donating 5-amino group, provide the large values of σ_p_ needed for maximal GSH induction. Additionally, the 5-amino group mitigates the toxicity that is associated a large σ_p_ value for the 4-position. As the values of DTT substituents cannot be increased much more without causing toxicity, it is likely that the activity of analog **6a** represents the upper limit of GSH induction for substituted DTTs.

**Fig. 6:**
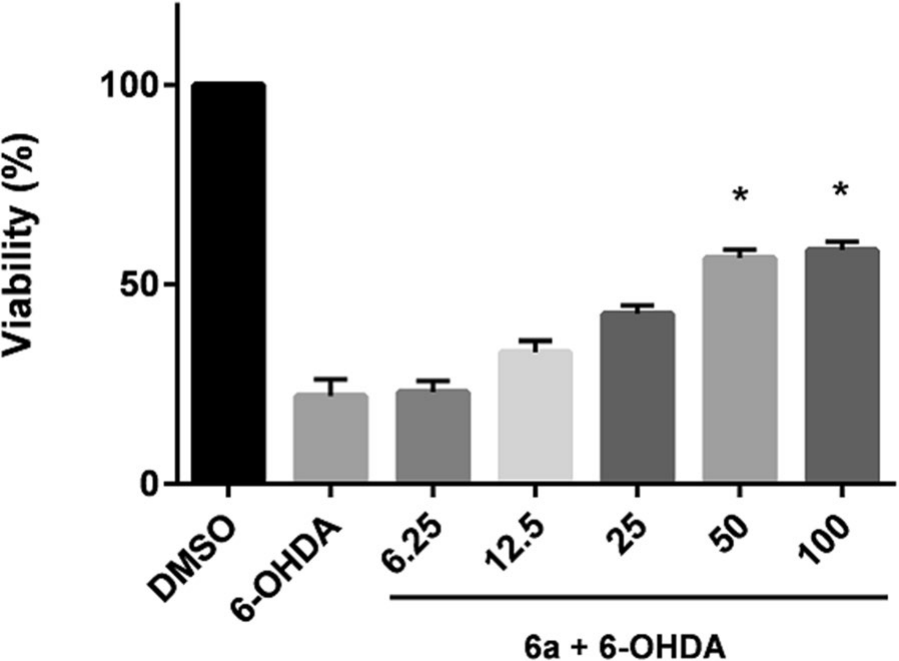
Neuroprotection of **6a** against 6-OH induced neurotoxicity. SH-SY5Y cells were treated for 24 h with various concentrations of **6a**, followed by co-treatment with 6-OHDA (40 μM) for a further 24 h, at which time cellular viability was assed. Data shown are mean ± SEM of at least three different experiments. *P < 0.05

Having identified a DTT that potently increases cellular GSH levels, we next evaluated the ability of **6a** to protect against 6-OHDA induced toxicity, a commonly used neuroprotection model [35–38]. SH-SY5Y cells were pretreated with **6a** for 24 h at concentrations of 6.25, 12.5, 25, 50, and 100 μM, followed by concurrent exposure to 40 μM 6-OHDA for a further 24 h. Cell viability was then determined. As shown in Fig. 6 administration of 40 µM 6-OHDA reduced cellular viability to 22 %. Excitingly, pretreatment with **6a** dose-dependently protected against the toxic effects of 6-OHDA. Protective effects were seen starting with a concentration of 12.5 µM (33 % viability), and plateaued with the doses of 50 and 100 µM; interestingly, these two doses were equally protective (56 and 58 %, respectively).

**Fig. 7:**
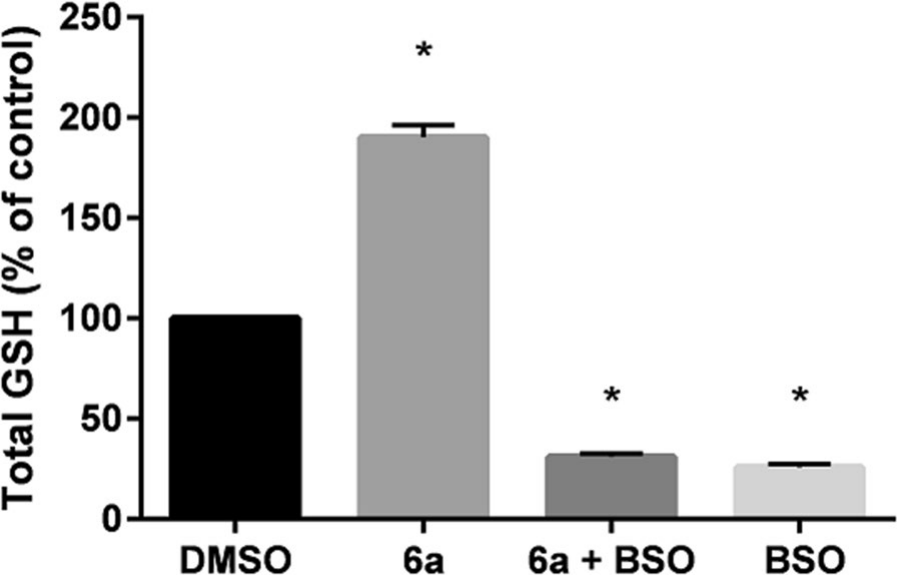
Suppression of GSH induction of **6a** by BSO. SH-SY5Y cells were treated with **6a** (100 μM) and/or BSO (25 μM) for 24 h, at which time total cellular GSH levels were assessed. Data shown are mean ± SEM of at least three different experiments. *P < 0.05

**Fig. 8:**
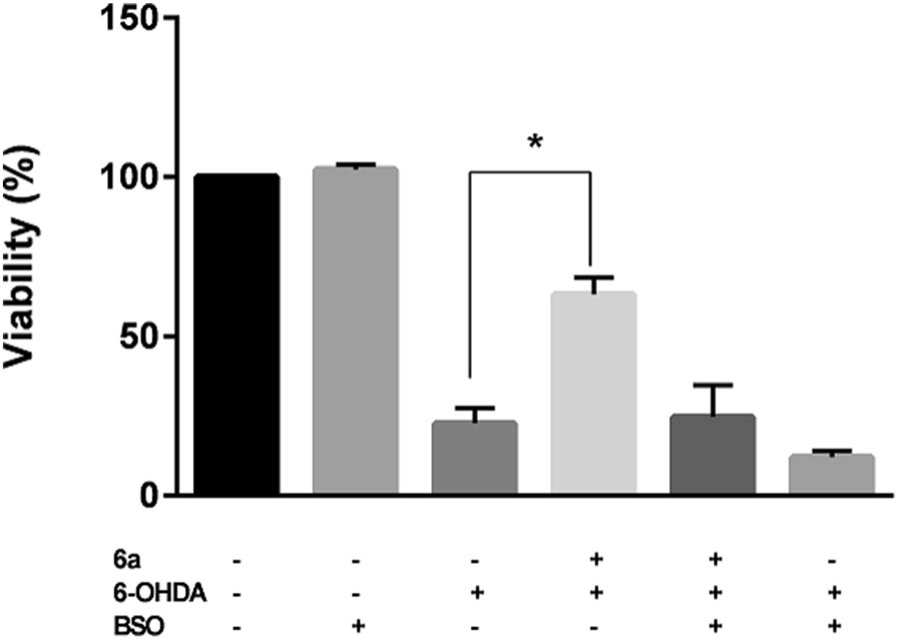
Abrogation of protective neuroprotective effects of **6a** by BSO. SH-SY5Y cells were treated with **6a** (100 μM) and/or BSO (25 μM) for 24 h, at which time either 6-OHDA (40 μM) or DMSO was added. Cellular viability was measured 24 h later. Data shown are mean ± SEM of at least three different experiments. *P < 0.05

The mechanism of 6-OHDA toxicity involves the generation of ROS and electrophilic quinone metabolites [39]. The increase in cellular GSH levels mediated by **6a** likely protects against the oxidative insult of 6-OHDA. To explore the role that GSH plays in this protection, SH-SY5Y cells were co-treated with **6a** and buthionine sulfoximine (BSO), an inhibitor of GCLC [40]. As shown in Fig. 7, administration of BSO (25 µM) was able to inhibit the ability of **6a** (100 µM) to induce GSH, demonstrating that GSH induction is mediated through actions of GCLC. Additionally, the abrogation of GSH induction by BSO was able to block the neuroprotective effects of **6a** (Fig. 8), confirming the importance of GSH in neuroprotection. DTTs are known, via stabilization of Nrf2, to induce the expression of numerous cytoprotective phase II enzymes, and it is possible that the activity of these enzymes contribute to the protective effects of **6a**. However, as the protective effects of **6a** can be blocked by inhibition of GSH induction, the contribution to neuroprotection of other phase II enzymes in this model may be minimal.

Many of the symptoms of PD arise as a result of depletion of nigrostriatal DA levels. As such, current antiparkinsonian pharmacotherapeutic approaches are DA focused. These treatments aim to replace DA (levodopa), slow its metabolism (inhibitors of monoamine oxidase B and catecholamine *O*-methyltransferase), or supplement its effects (dopamine agonists). While these agents are able to provide symptomatic relief in PD, they do little to halt or reverse the progression of the disorder since they do not address the underlying oxidative damage that is responsible for the loss of dopaminergic neurons. The results of this study, while preliminary, suggest that elevation of cellular levels of GSH may have promise as a potential antioxidant-based antiparkinsonian approach. Additional studies are currently planned to examine the neuroprotective potential of DTTs is additional cell lines and PD models.

## Conclusions

In support of our effort to identify novel potential neuroprotective agents, a further series of substituted DTTs was synthesized and evaluated for GSH induction in the SH-SY5Y human neuroblastoma cell line. Our results showed that the extent of GSH induction is related to the electronic properties of DTTs. Plots of GSH induction vs. DTT substituent Hammett σ_p_ values demonstrated linear relationships for substituents of 4-, 5-, and 4, 5-disubstituted DTTs. It was also observed that the magnitude of σ_p_ at the 4-position influences DTT toxicity, which can be diminished by the presence of an EDG at the 5-position. The most potent inducer of GSH identified in this study, congener **6a**, was minimally toxic to cells and was able to provide neuroprotection in the 6-OHDA model of neurotoxicity, suggesting GSH induction as a neuroprotective strategy. GSH induction was shown to be crucial to neuroprotection, as the protective effects of **6a** were abrogated by treatment with the GCLC inhibitor, BSO. The data generated in this study suggest that dithiolethiones warrant additional exploration as potential neuroprotective, antiparkinsonian agents.

## Experimental section

### Chemistry methods

All solvents and reagents obtained from commercial sources were used without further purification, unless otherwise noted. Compounds **6a** and **6f** were purchased from Oakwood Chemical (West Columbia, SC) and purified prior to use. All reactions were carried out under an argon atmosphere unless otherwise noted. All final molecules were >95 % pure as judged by high-performance liquid chromatography (HLPC). HPLC analyses were performed on an Agilent 1220 Infinity system with an Agilent column (Poroshell 120 EC-C18, 4.6 × 150 mm, gradient of 0.1 % trifluoroacetic acid/acetonitrile). ^1^H and ^13^C NMR analyses were performed on a Varian Mercury 300 MHz spectrophotometer at 300 and 75 MHz, respectively. Chemical shifts are given in ppm in reference to tetramethylsilane (TMS) as an internal standard. Multiplicities are given as s (singlet), d (doublet), t (triplet), m (multiplet), and br s (broad signal). Low-resolution mass spectral data were obtained on an Agilent 1260 Infinity single quadrupole LCMS system. Melting points were taken on a Mel-Temp apparatus and are uncorrected. Thin layer chromatography (TLC) was performed on silica gel 60 F_254_-coated glass plates purchased from EMD Millipore, and visualized with UV light and/or basic KMnO_4_.

### General procedure for the synthesis of dithiolethiones from β-keto esters, exemplified by 5-methyl-3H-1,2-dithiole-3-thione, **5a** [41]

To a suspension of elemental sulfur (123 mg, 3.85 mmol), phosphorus pentoxide (1.03 g, 2.31 mmol), hexamethyldisiloxane (2.76 mL, 11.6 mmol), in toluene (10 mL) was added β-oxo ester **2a** (500 mg, 3.85 mmol). The mixture was heated under reflux conditions until complete as judged by TLC (generally between 1 and 3 h), at which time the reaction mixture was cooled to 0 °C. Saturated aqueous K_2_CO_3_ was added (5 mL) to destroy any unreacted phosphorus pentoxide. The crude product was then extracted with ethyl acetate (10 mL × 3), dried (Na_2_SO_4_), filtered, concentrated, and purified by column chromatography (hexanes/ethyl acetate, 4:1) to give a low-melting red solid (521 mg, 91 %). R_*f*_ = 0.65 (20 % EtOAc/Hex). ^1^H NMR (300 MHz, CDCl_3_): δ 2.52 (d, *J* = 0.99 Hz, 3 H), 7.00–7.07 (m, 1 H). ^13^C NMR (75 MHz, CDCl_3_) δ: 18.43, 139.41, 172.22, 216.66. Calc. 148, found 149 [M+H]^+^.

### 4-(4-Nitrophenyl)-3H-1,2-dithiole-3-thione, **4a** [42]

Prepared from **1a** [43]. Red solid (92 %). Mp 152–154 °C. R_*f*_ = 0.37 (20 % EtOAc/Hex). ^1^H NMR (300 MHz, CDCl_3_): δ 7.89 (d, *J* = 8.73 Hz, 2 H), 8.30 (ds, *J* = 8.90 Hz, 2 H), 9.34 (s, 1 H). ^13^C NMR (75 MHz, CDCl_3_): δ = 128.67, 135.59, 145.44, 151.15, 152.50, 166.47, 218.57. Calc. 255, found 256 [M+H]^+^.

### 4-Ethyl-3H-1,2-dithiole-3-thione, **4b** [44]

Prepared from **1b** [45]. Yellow oil (81 %). R_*f*_ = 0.46 (20 % EtOAc/Hex). ^1^H NMR (300 MHz, CDCl_3_): δ 1.15 (t, *J* = 7.43 Hz, 3 H), 2.48–2.73 (m, 2 H), 8.86 (t, *J* = 0.82 Hz, 1 H). ^13^C NMR (75 MHz, CDCl_3_): δ 13.03, 23.52, 150.79, 155.45, 215.12. Calc. 162, found 163 [M+H]^+^.

### Ethyl 3-thioxo-3H-1,2-dithiole-4-carboxylate, **4c** [46]

Prepared from diethyl 2-(ethoxymethylene)malonate, **1c**. Red solid (47 %). Mp 61–62 °C. R_*f*_ = 0.48 (20 % EtOAc/Hex). ^1^H NMR (300 MHz, CDCl_3_): δ 1.37 (t, *J* = 7.07 Hz, 3 H), 4.35 (q, *J* = 7.19 Hz, 2 H), 9.18 (s, 1 H). ^13^C NMR (75 MHz, CDCl_3_): δ 14.35, 62.12, 138.30, 160.81, 165.22, 211.31. Calc. 207, found 208 [M+H]^+^.

### 5-(4-Fluorophenyl)-3H-1,2-dithiole-3-thione, **5b** [47]

Red solid (74 %). Mp 98–100 °C. R_*f*_ = 0.84 (20 % EtOAc/Hex). ^1^H NMR (300 MHz, CDCl_3_): δ 7.12–7.26 (m, 2 H) 7.39 (s, 1 H) 7.59–7.72 (m, 2 H). ^13^C NMR (75 MHz, CDCl_3_): δ 116.97/117.26 (CF, d, *J* = 22 Hz), 129.19, 129.31, 136.13, 163.45/166.83 (CF, d, *J* = 254 Hz), 171.62, 215.66. Calc. 228, found 229 [M+H]^+^.

### 5-(Pyridin-4-yl)-3H-1,2-dithiole-3-thione, **5c** [48]

Red solid (34 %). Mp decomposed. R_*f*_ = 0.09 (20 % EtOAc/Hex). ^1^H NMR (300 MHz, CDCl_3_): δ 7.50 (s, 1 H) 7.52–7.59 (m, 2 H) 8.81 (d, *J* = 5.93 Hz, 2 H). ^13^C NMR (75 MHz, CDCl_3_): δ 121.02, 121.9, 145.67, 150.01, 175.25, 214.27. Calc. 211, found 212 [M+H]^+^.

### 5-(Furan-2-yl)-3H-1,2-dithiole-3-thione, **5d** [49]

Red solid (63 %). Mp 97–100 °C. R_*f*_ = 0.71 (20 % EtOAc/Hex). ^1^H NMR (300 MHz, CDCl_3_): δ 6.61 (dd, *J* = 3.53, 1.72 Hz, 1 H), 6.95–7.02 (m, 1 H), 7.38 (s, 1 H), 7.64 (dd, *J* = 1.81, 0.54 Hz, 1 H). ^13^C NMR (75 MHz, CDCl_3_): δ 113.53, 113.59, 133.27, 146.60, 146.71, 160.27, 214.50. Calc. 200, found 201 [M+H]^+^.

### Ethyl 5-methyl-3-thioxo-3H-1,2-dithiole-4-carboxylate, **6b** [50]

Red solid (78 %). Mp 64–66 °C. R_*f*_ = 0.84 (20 % EtOAc/Hex). ^1^H NMR (300 MHz, CDCl_3_): δ 1.37 (t, *J* = 7.16 Hz, 3 H), 2.57 (s, 3 H), 4.39 (q, *J* = 7.07 Hz, 2 H). ^13^C NMR (75 MHz, CDCl_3_): δ 14.35, 19.11, 62.50, 140.80, 163.28, 174.05, 211.82. Calc. 220, found 221 [M+H]^+^.

### 4-Chloro-5-(4-methoxyphenyl)-3H-1,2-dithiole-3-thione, **6g** [51]

Prepared from **3g** [52]. Yellow solid (91 %). Mp 125–127 °C. R_*f*_ = 0.63 (20 % EtOAc/Hex). ^1^H NMR (300 MHz, CDCl_3_): δ 3.90 (s, 3 H), 7.07 (d, *J* = 9.06 Hz, 2 H), 7.67 (d, *J* = 9.06 Hz, 2 H). ^13^C NMR (75 MHz, CDCl_3_): δ 55.57, 114.78, 124.12, 130.39, 123.43, 162.45, 165.62, 206.59. Calc. 274, found 275 [M+H]^+^.

### 4-Chloro-5-phenyl-3H-1,2-dithiole-3-thione, **6h** [51]

Prepared from **3h**  [53]. Yellow solid (87 %). Mp 105–107 °C. R_*f*_ = 0.74 (2 % EtOAc/Hex). ^1^H NMR (300 MHz, CDCl_3_): δ 7.49–7.73 (m, 5 H). ^13^C NMR (75 MHz, CDCl_3_): δ 127.07, 128.88, 129.49, 129.79, 131.91, 165.63, 206.88. Calc. 244, found 245 [M+H]^+^.

### 4-Chloro-5-ethyl-3H-1,2-dithiole-3-thione, **6i** [54]

Prepared from **3i** [55]. Yellow solid (59 %). Mp 83–84 °C. R_*f*_ = 0.71 (20 % EtOAc/Hex). ^1^H NMR (300 MHz, CDCl_3_): δ 1.40 (t, *J* = 7.52 Hz, 3 H), 2.98 (q, *J* = 7.61 Hz, 2 H). ^13^C NMR (75 MHz, CDCl_3_): δ 12.80, 27.99, 158.84, 171.46, 206.64. Calc. 196, found 197 [M+H]^+^.

### Ethyl 5-acetamido-3-thioxo-3H-1,2-dithiole-4-carboxylate, **6c** [56]

Compound **6a** (100 mg, 0.452 mmol) was refluxed in acetic anhydride (5 mL) for 30 min. The solution was then cooled, concentrated to dryness, and the crude material purified by column chromatography (hexanes/ethyl acetate, 3:1) to give **6c** as a red solid (104 mg, 88 %). Mp 156–157 °C. R_*f*_ = 0.39 (20 % EtOAc/Hex). ^1^H NMR (300 MHz, CDCl_3_): δ 1.43 (t, *J* = 7.16 Hz, 3 H), 2.40 (s, 3 H), 4.42 (q, *J* = 7.13 Hz, 2 H), 12.72 (br s, 1 H). ^13^C NMR (75 MHz, CDCl_3_): δ 14.15, 23.97, 62.68, 118.75, 166.36, 170.63, 174.56, 208.25. Calc. 263, found 264 [M+H]^+^.

### General procedure for the syntheses of dithiolethiones from nitriles, exemplified by 5-amino-4-(4-chlorophenyl)-3H-1,2-dithiole-3-thione, **6d**

To an ice-cooled suspension of NaH (263 mg, 6.58 mmol), carbon disulfide (220 μL, 3.62 mmol), and elemental sulfur (116 mg, 3.62 mmol) in DMF (5 mL) was added **3d** (500 mg, 3.29 mmol) in DMF (1 mL). The mixture was allowed to stir at 0 °C for 30 min, at which time saturated Na_2_CO_3_ (10 mL) was added. The mixture was then extracted with ethyl acetate (10 mL × 3), washed with water (10 mL × 3), dried (Na_2_SO_4_), filtered, concentrated, and purified by column chromatography (hexanes/ethyl acetate 4:1) to yield **6d** as a red solid (838 mg, 95 %). Mp 106–107 °C. R_*f*_ = 0.29 (20 % EtOAc/Hex). ^1^H NMR (300 MHz, CDCl_3_): δ 6.35 (br s, 2 H), 7.29 (d, *J* = 8.70 Hz, 2 H), 7.48 (d, *J* = 8.70 Hz, 1 H). ^13^C NMR (75 MHz, CDCl_3_): δ 130.00, 132.22, 132.27, 134.85, 151.04, 175.69, 234.84. Calc. 259, found 260 [M+H]^+^.

### 5-Amino-4-(phenylsulfonyl)-3H-1,2-dithiole-3-thione, **6e** [30]

Red solid (69 %). Mp decomposed. R_*f*_ = 0.13 (20 % EtOAc/Hex). ^1^H NMR (300 MHz, CDCl_3_): δ 7.50–7.78 (m, 3 H), 7.91–8.05 (m, 2 H), 9.01 (bs 1 H), 10.09 (bs, 1 H). ^13^C NMR (75 MHz, CDCl_3_): δ 117.75, 127.39, 128.77, 133.74, 140.45, 180.23, 203.60. Calc. 289, found 290 [M+H]^+^.

### Biological methods

#### Cell culture conditions

The SH-SY5Y human neuroblastoma cell line was obtained from the American Type Culture Collection (ATCC, Manassas, VA). Cells were grown in DMEM:F-12 media (1:1) supplemented with FBS (10 %) and 100 U/mL penicillin and 100 μg/mL streptomycin in 150 cm^2^ culture flasks in a humidified atmosphere of 5 % CO_2_. The media was replaced every 3–4 days, and cells were subcultured once a confluence of 70–80 % was reached. All test compounds were dissolved in DMSO and diluted in media (final DMSO concentration of 0.1 % v/v).

### Measurement of intracellular GSH levels

SH-SY5Y cells were seeded in white 96-well plates and allowed to adhere overnight. Media was removed and replaced with media containing either test compounds (100 μM) or DMSO (0.1 %) for 24 h. Total glutathione levels (GSH + GSSG) were then measured using GSH/GSSG Glo© assay from Promega (Madison, WI). GSH levels were expressed as a percentage of control.

### Neuroprotection assay

SH-SY5Y cells were seeded in white 96 well plates and allowed to attach overnight. Media was removed and replaced with media containing either test compounds (100 μM) or DMSO for 24 h. Next, 6-OHDA (Aldrich) in media (final concentration of 40 μM) of media was added and the cells were co-treated for 24 h. Cellular viability was assessed using the CellTiter Glo© assay from Promega (Madison, WI). Viability was expressed as a percentage of control.

### Statistical analyses

One-way analysis of variance (ANOVA) was used to test for significant differences using GraphPad Prism software (La Jolla, CA). *P* values less than 0.05 were considered to be statistically significant. Results are expressed as mean ± SEM.

## Abbreviations

6-OHDA: 6-hydroxydopamine
ARE: antioxidant response element
BSO: buthionine sulfoximine
DA: dopamine
DTTs: dithiolethiones
EDGs: electron donating groups
EWGs: electron withdrawing groups
GCL: glutamate cysteine ligase
GPx: glutathione peroxidase
GSH: glutathione
GSSG: oxidized glutathione
GST: glutathione *S*-transferase
Keap1: Kelch-like ECH-associated protein-1
NQO1: NAD(P)H:quinone oxidoreductase
Nrf2: nuclear factor-erythroid-2 related factor-2
PD: Parkinson’s disease
ROS: reactive oxygen species
